# Effect of Intergenerational Physiotherapy on Functional Mobility and Quality of Life in Patients With Parkinson’s Disease

**DOI:** 10.7759/cureus.108779

**Published:** 2026-05-13

**Authors:** Sakshi P Kavitake, Suraj Kanase

**Affiliations:** 1 Department of Neuroscience, Krishna College of Physiotherapy, Krishna Vishwa Vidyapeeth (Deemed to be University), Karad, IND

**Keywords:** intergenerational physiotherapy, motivation, parkinson’s disease, psychosocial wellbeing, quality of life

## Abstract

Introduction

Parkinson’s disease (PD) leads to progressive motor symptoms such as bradykinesia, rigidity, tremors, and postural instability, along with non-motor symptoms that reduce quality of life. Physiotherapy helps improve mobility, balance, and independence, but conventional approaches may suffer from low motivation and poor adherence. Intergenerational physiotherapy (IGP) includes pairing individuals with PD with younger partners to enhance engagement, provide social interaction, and introduce natural dual-task challenges. This supportive environment can improve gait, balance, posture, mood, and confidence. Although promising, IGP is still under-researched. This study investigated the physical and psychosocial benefits of Parkinson’s rehabilitation.

Methods

A six-month randomized controlled trial was conducted at Krishna College of Physiotherapy with 18 individuals diagnosed with idiopathic PD. Participants were randomly allocated to either IGP or conventional physiotherapy. Eligible adults aged ≥50 years underwent supervised sessions five times per week for four weeks. Individuals with unstable medical conditions were excluded. Standardized assessments were used to measure motor performance, balance, gait parameters, and overall quality of life before and after the intervention.

Results

The results of the study demonstrated significant improvements in the IGP group compared with the conventional physiotherapy group across multiple parameters. Both groups showed progress after intervention; however, the IGP group exhibited greater gains in mobility, balance, and quality of life. Timed Up and Go scores, PD questionnaire-39 scores, and Berg Balance Scale scores improved more significantly in the IGP group, with p-values < 0.005, indicating high statistical significance. No significant differences were observed between groups at baseline, confirming group comparability. Overall, IGP proved more effective in enhancing functional performance and psychosocial well-being in individuals with PD.

Conclusion

Participants with PD showed positive responses to both conventional and IGP, with the latter demonstrating added physical and psychosocial benefits. These findings emphasize the potential of IGP as an engaging and holistic rehabilitation approach. Incorporating social interaction and motivation through younger partners may enhance adherence, functional outcomes, and overall quality of life in individuals with PD.

## Introduction

Parkinson’s disease (PD) is a progressive neurodegenerative disorder primarily affecting the dopaminergic neurons in the substantia nigra, leading to a gradual decline in motor control and coordination [1]. It is the second most common neurodegenerative disorder worldwide after Alzheimer’s disease, with prevalence increasing significantly with age [2]. The cardinal motor symptoms of PD include bradykinesia, rigidity, tremors, and postural instability [3], which collectively impair mobility and functional independence [4]. In addition to motor manifestations, PD is also associated with a wide range of non-motor symptoms, including cognitive impairment, depression, anxiety, sleep disturbances, fatigue, and autonomic dysfunction, all of which can significantly affect patients’ quality of life [5]. These symptoms often appear early in the disease course and substantially contribute to disability, reduced participation in daily activities, and poor quality of life [6]. Physiotherapy plays a crucial role in the multidisciplinary management of PD by addressing both motor and functional impairments [7]. Physiotherapy interventions aim to improve gait, posture, balance, flexibility, and overall physical conditioning [8]. Conventional physiotherapy techniques include gait training, balance exercises, strength training, and cueing strategies that help overcome movement initiation difficulties [9]. Although these interventions are clinically effective, their long-term success is often limited by poor motivation, reduced adherence, and social isolation among patients [10]. Sustained participation in therapy requires engagement, emotional support, and a sense of purpose, factors that are sometimes overlooked in traditional rehabilitation settings [11]. Intergenerational physiotherapy (IGP) has recently emerged as an innovative approach that integrates physical rehabilitation with social engagement [12]. It involves pairing older adults with PD with younger participants, such as adolescents or young adults, in structured physiotherapy sessions. The younger partners serve as exercise companions, motivators, and sources of enthusiasm, fostering a supportive and interactive environment [13]. This model not only enhances physical performance through playful, dual-task, and motivational activities but also strengthens psychological well-being by reducing feelings of loneliness and social withdrawal [14]. Additionally, younger participants benefit through increased empathy, understanding, and awareness of aging and neurological disorders [15]. Despite the potential benefits of IGP, there is limited evidence regarding its effects on functional mobility and quality of life in patients with PD. Therefore, this study aims to evaluate the effect of IGP on these outcomes.

## Materials and methods

This experimental study with a randomized controlled trial design was conducted at Krishna College of Physiotherapy, Krishna Vishwa Vidyapeeth, Karad, over a period of six months. A total of 18 participants diagnosed with idiopathic PD were selected using a random sampling method. Participants were randomly assigned to two groups: an experimental group receiving IGP along with conventional physiotherapy, and a control group receiving conventional physiotherapy. Randomization was performed using a random number table to generate the allocation sequence, and allocation concealment was maintained using a centralized method. Due to the nature of the intervention, blinding of the participants and treating therapists was not feasible. However, the outcome assessor and data-entry operator were blinded to group allocation to minimize assessment and analysis bias during the study.

Inclusion and exclusion criteria

Individuals aged 50-75 years, diagnosed with idiopathic PD by a neurologist, and with a disease duration of at least one year were included in the study, as the severity and progression of symptoms may vary with disease duration. Participants on stable antiparkinsonian medication with mild-to-moderate disability (Hoehn and Yahr stages I-III), who were able to walk at least 10 m independently or with one assistive device, willing to provide informed consent, and able to attend regular sessions were also included. Participants with unstable cardiac or pulmonary disease, severe uncorrected visual or hearing impairment, cognitive deficits affecting comprehension and participation, severe musculoskeletal conditions limiting mobility, or individuals at a high risk of falls were excluded from the study. 

This study was registered with the Clinical Trials Registry of India under the registration number CTRI/2026/04/109686. A formal sample size calculation was not performed, as this was an exploratory study conducted within a limited study duration and based on participant availability, which has been acknowledged as a limitation of the study.

The intervention for the experimental group involved structured IGP sessions conducted with younger partners aged 18-25 years who were medically fit, able to participate in physical activity, and willing to attend regular sessions, incorporating exercises for balance, gait, coordination, and functional mobility.

Data source and tools

The control group performed conventional physiotherapy, including gait training, balance exercises, strength training, and cueing strategies. Both groups attended therapy sessions five times per week for four weeks, each lasting approximately 45-60 minutes under professional supervision. Outcome measures included the Timed Up and Go scores, Berg Balance Scale scores, and PD questionnaire-39 scores to assess motor function, balance, gait, and quality of life, respectively. Data were collected before and after the intervention, statistically analyzed, and presented in a summarized report upon study completion.

The intervention was conducted over a period of four weeks, with participants allocated to two groups: an experimental group (Group A) receiving IGP and a control group (Group B) receiving conventional physiotherapy. Each session lasted approximately 40-45 minutes and was performed under supervision. In Week 1, both groups began with a seven-minute warm-up consisting of low-intensity activities such as seated marching, arm movements, and breathing exercises. Group A additionally incorporated child-assisted interactions during exercises. This was followed by the main training phase, where Group A performed a structured functional circuit, including sit-to-stand, 10-m walking with external cueing, balance training (weight shifts and tandem stance), and task-oriented activities such as ball passing. Group B performed similar functional tasks independently, along with ankle range-of-motion exercises. Group A further engaged in dual-task and social interaction activities, including cognitive-motor tasks and rhythm-based exercises, which were not included in Group B. Both groups concluded with a cool-down phase involving stretching and breathing exercises.

In Week 2, progression was introduced by increasing repetitions and task complexity. Group A performed sit-to-stand (3×10 repetitions), step-up and step-down tasks, obstacle negotiation, and resistance-based exercises integrated with partner interaction. Dual-task training included combined cognitive and motor activities such as category naming during ambulation. Group B performed comparable exercises without social or dual-task components, including fast walking and balance training on unstable surfaces.

In Week 3, further progression included dynamic and functional challenges. Group A performed sit-to-stand with object carrying, obstacle courses, reactive stepping, and interactive ball activities, along with cognitive dual-task training such as memory sequencing and backward counting during walking. Group B performed similar motor tasks, including object transfer, obstacle navigation, and tandem walking, without interactive or cognitive integration.

In Week 4, both groups performed advanced functional training. Group A engaged in a mini-circuit combining sit-to-stand, fast walking, and step-up tasks, along with tandem walking and continuous walking for endurance, with motivational input from child partners. Additional components included group-based activities such as relay tasks and rhythm exercises to enhance engagement and social participation. Group B performed structured functional and endurance training, including mini-circuits, six-minute walk practice, tandem stance, and single-leg reach activities. All sessions in both groups concluded with standardized cool-down protocols consisting of stretching of major muscle groups and relaxation breathing exercises.

This structured progression ensured a gradual increase in intensity, complexity, and functional relevance, with the key distinction being the incorporation of intergenerational interaction and dual-task training in the experimental group.

Statistical analysis

All data were entered into a Microsoft Excel (Microsoft Corp., Redmond, WA, USA) spreadsheet and analyzed using GraphPad InStat software (version 3.06, trial version, GraphPad Software, Inc., San Diego, CA, USA). Descriptive statistics, including mean and SD, were calculated for all outcome measures. A paired t-test was used to assess within-group differences between pre- and post-intervention values. An unpaired t-test was used to compare differences between the experimental and control groups. CIs (95% CI) and effect sizes were calculated for key outcome measures to assess the precision and magnitude of the treatment effects. A p-value of less than 0.05 was considered statistically significant.

## Results

Table 1 presents the gender distribution of the study participants, including the frequencies and percentages of males and females.

**Table 1:** Gender distribution of the study participants

Gender	Frequency (n)	Percentage (%)
Male	10	55.6
Female	8	44.4
Total	18	100

The comparison of pre-test and post-test scores between Group A (IGP) and Group B (conventional physiotherapy) is presented in Table 2. At baseline, there was no statistically significant difference between the two groups (p=0.797). However, post-intervention analysis demonstrated a statistically significant improvement in Group A compared with Group B (p = 0.004). Furthermore, the mean improvement observed in Group A was significantly greater than that of Group B (p=0.001), indicating the superior effectiveness of IGP.

**Table 2:** Comparison of Timed Up and Go scores between Group A and Group B

Parameter	Group A (intergenerational physiotherapy)	Group B (conventional physiotherapy)	Mean difference (A–B)	t-value	p-value	Significance
Pre-test mean ± SD	14.82 ± 1.96	15.03 ± 2.15	-0.21	0.26	0.797	Not significant
Post-test mean ± SD	10.43 ± 1.52	12.87 ± 1.84	-2.44	3.26	0.004	Significant
Mean improvement (pre–post)	4.39 ± 0.95	2.16 ± 1.02	2.23	4.11	0.001	Highly significant

The comparison of outcome measures between Group A (IGP) and Group B (conventional physiotherapy) is presented in Table 3. At baseline, no statistically significant difference was observed between the groups (p=0.584), indicating that both groups were comparable before the intervention. Post-intervention analysis revealed that Group A demonstrated significantly lower scores than Group B (p=0.003), reflecting greater improvement following IGP. Furthermore, the mean improvement in Group A was substantially higher than that observed in Group B, with the difference being highly statistically significant (p=0.001). These findings suggest that IGP was more effective in enhancing outcomes compared with conventional physiotherapy.

**Table 3:** Comparison of quality of life (PDQ-39) scores between Group A and Group B PDQ-39, Parkinson’s disease questionnaire-39.

Parameter	Group A (intergenerational physiotherapy)	Group B (conventional physiotherapy)	Mean difference (A–B)	t-value	p-value	Significance
Pre-test mean ± SD	62.78 ± 5.46	61.35 ± 6.02	1.43	0.56	0.584	Not significant
Post-test mean ± SD	45.11 ± 4.93	52.64 ± 5.11	-7.53	3.42	0.003	Significant
Mean improvement (pre–post)	17.67 ± 3.24	8.71 ± 2.98	8.96	4.88	0.001	Highly significant

The comparative evaluation of outcome measures between Group A (IGP) and Group B (conventional physiotherapy) is presented in Table 4. At baseline, no statistically significant difference was observed between the groups (p=0.753), indicating initial comparability. Following the intervention, Group A demonstrated significantly higher post-test scores than Group B (p=0.004), suggesting a greater therapeutic benefit associated with IGP. Moreover, the mean improvement in Group A was considerably greater than that observed in Group B, with the difference highly statistically significant (p=0.001). These results further support the superior effectiveness of IGP over conventional approaches.

**Table 4:** Comparison of Berg Balance Scale scores between Group A and Group B

Parameter	Group A (intergenerational physiotherapy)	Group B (conventional physiotherapy)	Mean difference (A–B)	t-value	p-value	Significance
Pre-test mean ± SD	38.2 ± 3.1	37.8 ± 3.4	0.4	0.32	0.753	Not significant
Post-test mean ± SD	46.7 ± 2.8	41.2 ± 3.0	5.5	3.45	0.004	Significant
Mean improvement (pre–post)	8.5 ± 1.6	3.4 ± 1.5	5.1	4.12	0.001	Highly significant

## Discussion

This study investigated the comparative effects of IGP and conventional physiotherapy in individuals with idiopathic PD [16]. The findings demonstrated greater improvements in gait, balance, coordination, and quality of life in the IGP group than the conventional physiotherapy group [17]. These enhanced outcomes may be associated with the combined influence of physical activity, social interaction, cognitive engagement, and motivational support integrated within the rehabilitation process [18]. PD is frequently associated with reduced motivation, depression, apathy, and social isolation, which can negatively affect participation and long-term adherence to rehabilitation programs [19]. The inclusion of younger partners in IGP may have increased emotional engagement, confidence, and active participation by creating a more supportive and interactive therapeutic environment. Another possible mechanism underlying the observed improvements may be the incorporation of dual-task activities involving simultaneous cognitive and motor performance. Previous studies have suggested that cognitive-motor training may enhance neuroplasticity, improve motor learning, and promote better postural control and gait performance in individuals with PD [20]. In the present study, activities such as obstacle negotiation, rhythm-based exercises, interactive ball activities, and cognitive-motor tasks may have stimulated attentional control, coordination, and movement initiation, thereby contributing to improved functional mobility and balance outcomes. Conventional physiotherapy is well established in improving mobility, muscle strength, balance, and functional independence in PD [21]. However, many conventional rehabilitation approaches primarily focus on motor symptoms and may inadequately address psychosocial and emotional aspects of patient care [22]. The present findings are consistent with previous literature demonstrating that socially engaging and group-based rehabilitation strategies can positively influence both physical and psychological outcomes in neurological populations [23]. Similar improvements in motivation, participation, and emotional well-being have been reported in studies involving dance therapy, interactive exercise programs, and group-based physiotherapy interventions in individuals with PD and older adults [24]. However, unlike several previously reported interventions, the current study incorporated structured intergenerational interaction alongside physiotherapy exercises, which may explain the greater improvements observed in the IGP group. The psychosocial benefits observed in the present study are clinically important because non-motor symptoms significantly affect quality of life and functional independence in individuals with PD [25]. Participants receiving IGP demonstrated improved confidence, emotional well-being, and feelings of connectedness, which may contribute to greater self-efficacy and participation in daily activities [26]. In addition, reciprocal interaction between older adults and younger participants may have fostered empathy, emotional support, and reduced feelings of loneliness and social withdrawal [27]. These findings support the growing emphasis on holistic and person-centered rehabilitation approaches in neurological physiotherapy. Despite these encouraging findings, certain limitations should be considered while interpreting the results. The relatively small sample size, absence of a formal sample size calculation, short intervention duration, and single-center study design may limit the generalizability of the findings [28]. Furthermore, the lack of long-term follow-up restricts conclusions regarding the sustainability of treatment effects over time [29]. Future multicenter studies with larger sample sizes and extended follow-up periods are recommended to further evaluate the long-term effectiveness of IGP and to better understand the individual contribution of social interaction, cognitive engagement, and exercise intensity to rehabilitation outcomes [30]. Overall, IGP appears to be a promising adjunct to conventional rehabilitation in PD by addressing both physical and psychosocial aspects of patient care.

## Conclusions

Based on data collected from participants with PD undergoing physiotherapy, it is evident that IGP offers promising benefits in both physical and psychosocial domains. The study showed that patients receiving IGP demonstrated better motivation, improved adherence, and enhanced functional outcomes such as gait, balance, and coordination compared to those receiving conventional therapy. This suggests a solid foundation of acceptance and engagement toward this innovative approach. Participants expressed positive responses regarding the emotional and social support provided through interaction with younger partners, reporting improvements in mood, confidence, and feelings of connectedness. These findings highlight the importance of addressing non-motor symptoms alongside physical rehabilitation in PD. Moreover, the younger participants showed enthusiasm and gained empathy, indicating mutual benefits across generations. Despite these encouraging results, some challenges were noted such as logistical difficulties in session scheduling, a relatively small sample size, and the absence of a formal sample size calculation, which may limit the broader applicability of the findings. Nevertheless, the overall positive response underscores the potential of IGP as an effective complementary strategy to enhance the quality of life for people living with PD. Further research with larger cohorts and longer follow-up is recommended to confirm these benefits and address practical barriers to implementation. Continued development and support for IGP could play a crucial role in improving both the physical and psychosocial well-being of this population.
